# Primary prevention of psychosis through interventions in the symptomatic prodromal phase, a pragmatic Norwegian Ultra High Risk study

**DOI:** 10.1186/s12888-015-0470-5

**Published:** 2015-04-22

**Authors:** Inge Joa, Jens Gisselgård, Kolbjørn Brønnick, Thomas McGlashan, Jan Olav Johannessen

**Affiliations:** 1 Psychiatric Division, Stavanger University Hospital, TIPS- Centre for Clinical Research in Psychosis, Armauer Hansensvei 20, N-4011 Stavanger, Norway; 2 Network for medical sciences, University of Stavanger, 4036 Stavanger, Norway; 3 Department of Psychiatry, Yale University, New Haven, CT USA

**Keywords:** Psychosis, Schizophrenia, Prodromal, Ultra-high risk, Early detection

## Abstract

**Background:**

Evidence has been accumulating that it may be possible to achieve prevention in psychotic disorders. The aim of the Prevention Of Psychosis (POP) study is to reduce the annual incidence of psychotic disorders in a catchment area population through detection and intervention in the prodromal phase of disorder. Prodromal patients will be recruited through information campaigns modelled on the Scandinavian early Treatment and Intervention in Psychosis (TIPS) study and assessed by low-threshold detection teams.

**Methods/Design:**

The study will use a parallel control design comparing the incidence of first episode psychotic disorders between two Norwegian catchment areas with prodromal detection and treatment (Stavanger and Fonna) with two catchment areas without a prodromal intervention program (Bergen and Østfold). The primary aim of the current study is to test the effect of a Prodromal Detection and Treatment program at the health care systems level. The study will investigate: 1) If the combination of information campaigns and detection teams modelled will help in identifying individuals (age 13–65, fulfilling study inclusion criteria) at high risk of developing psychosis early, and 2) If a graded, multi-modal treatment program will reduce rates of conversion compared to the rates seen in follow-along assessments.

**Discussion:**

Positive results could potentially revolutionize therapy by treating risk earlier rather than disorder later and could open a new era of early detection and intervention in psychosis. Negative results will suggest that the potential for psychosis is determined early in life and that research should focus more on genetically linked neurodevelopmental processes.

If we can identify people about to become psychotic with high accuracy, we can track them to understand more about how psychosis unfolds. Appropriate intervention at this stage could also prevent or delay the onset of psychosis and/or subsequent deterioration, i.e., social and instrumental disability, suicide, aggressive behavior, affective- and cognitive deficits.

**Trial registration:**

Current Controlled Trials ISRCTN20328848. Registered 02 November 2014.

## Background

Mental illness is the largest cause of disability in developed countries, and psychotic disorders (schizophrenia/bipolar disorder) are ranked among the leading causes of total burden of disease and cost of lifetime disability worldwide. In spite of high research activity the mechanisms behind these disorders are still primarily unknown and the effects of available treatments are also limited and mostly palliative.

Most patients experience a relative long period with non-psychotic symptoms before their first psychotic episode characterized by unspecific- and more specific symptom constellations (prodrome/Ultra High Risk (UHR) state). At the onset of psychosis, many have already experienced a loss of cognitive- and psychosocial functioning. We now know that individuals at high risk for psychotic disorders can be identified before this point of time. The present study builds on experiences from the Scandinavian Early Treatment and Intervention in Psychosis (TIPS) study [1,2], where the use of information campaigns and detection teams were instrumental in reducing the duration of untreated psychotic symptoms in the first episode. This reduction was followed by a more benign course of the disorder over ten years of treatment [3]. We here hypothesize that a similar program (Prodromal Detection and Treatment program - PDT) - aimed at identifying and treating help seeking individuals at high risk for developing psychotic disorders will a) significantly increase the number of high risk individuals that get in contact with the relevant services and b) that these individuals will be detected at a very early stage of illness development. After contact the high risk individuals will be allocated to a structured follow-along by a designated case manager or to a graded, multi-modal and primarily psychosocial treatment intervention. Patients will be offered active antipsychotic medication only at imminent risk of conversion to psychosis. We also hypothesize that the multi-modal treatment intervention will significantly reduce the number of persons converting to psychosis, and thus influence the incidence of new cases of first-episode psychosis. The study is designed as a regional multi-center study, involving central research- and clinical psychiatric organizations across the Health Vest region in Southern Norway, with significant international cooperation, and will involve all service levels from general practitioners to specialized psychiatric services. Two Norwegian treatment centers covering a population of 440 000 will collaborate in recruiting prodromal patients to the study: Stavanger and Fonna hospitals. The project organization has additional involvement from the research departments at Bergen University Hospitals and Sykehuset Østfold Hospital for establishing a first-episode psychosis incidence registration as another part of this research initiative. All study enrolled patients will be asked for blood samples and to be a part of the Norwegian NASATS program – Severe Mental Illness, aiming to identify new genetic variants associated with severe mental disorders [4].

### Rationale

Schizophrenia and other psychotic disorders are serious, costly, and disabling. They typically emerge in late adolescence and early adulthood, a critical phase of neurological, psychological and social development. Even if the number of new cases per year is low (varying between 15 in the TIPS study [1] to −30 per 100 000 per year [5], their early onsets combined with a high risk of chronic symptoms makes these into disorders with a high prevalence (1%).

Many patients display significant impairments already at start of first treatment, and it is known that course and outcome are poorer in patients that come late into treatment. Results from the TIPS study show for the first time that treatment can be initiated significantly earlier in psychotic disorders. This is in turn associated with less severe psychotic symptoms, suicidality and functional disabilities already at first treatment [1,6]. Follow-up after two- and five years in treatment demonstrate long-term advantages in the form of less severe negative symptoms, cognitive deficits, depressive symptoms and global dysfunction [7,8] and for the level of recovery rates up to ten year follow up. Early detection and treatment thus appears to have reduced collateral damage in first psychosis and altered the course and prognosis of the disorder (i.e. achieved secondary prevention). Given these empirical data it is timely to test whether *very* early intervention can actually prevent the development of disorder in individuals at high risk (i.e. primary prevention).

The symptomatic “ultra-high-risk” (UHR) or “prodromal” state has been a part of schizophrenia. In the past decade it has also become a reliably identifiable clinical constellation, with clear power to predict the onset of psychotic disorder (schizophrenia and other psychotic disorders) within the near future. The field of UHR research has the potential to shed light on the development of major psychotic disorders and to alter their course. It also provides a rationale for service provision to those in need of help who could not previously access it and the possibility of changing trajectories for those with vulnerability to psychotic illnesses [9]. These UHR individuals exhibit one or more of the existing prodromal syndromes: The attenuated positive symptom (APS) syndrome and/or the genetic risk and deterioration (GRD) syndrome. The most commonly used instruments to assess prodromal states include those developed in Melbourne (Comprehensive Assessment of At-Risk Mental States (CAARMS)) [10] and New Haven (Structured Interview for Prodromal Syndromes (SIPS) [11]. Because the prodrome is clinical in nature, persons developing these symptoms often seek a familiar clinical caregiver such as a general practitioner, a school counselor or a friend in a healthcare position. Because the UHR state is a relatively rare and new clinical constellation not widely known, recruiting symptomatic, help-seeking persons at risk for psychosis requires active outreach to healthcare-oriented referral sources [12]. This includes educating referral sources about the prodrome, training and maintaining triage staff and conducting rapid, low threshold evaluations of referrals.

### Conversion to psychosis in prodromal samples

In studies using the New Haven/Melbourne UHR criteria, preliminary reports found a 40-50% annual rate of conversion to psychosis of identified individuals [13,14]. This is in line with studies using similar criteria, including studies from Cologne who found a conversion rate of 49 % after 9.6 years [15] and a London based study finding conversion rates of 50% after 2 years [16]. A later study from Melbourne found conversion rates of 34.6% in a sample of 104 UHR subjects identified with the CAARMS [17]. The more recent North American Prodromal Longitudinal Study (NAPLS) - found that 28% of the cases in the study converted to during follow-up. The rate of conversion showed a decelerating trend during the follow-up, with the majority of converters the first two years of the study [18]. At 2.5 year follow up a conversion rate of 29% is reported [19]. In the NAPLS sample, several variables predicted conversion; the most powerful were a diagnosis of schizotypal personality disorder, substance abuse, a recent decline in functioning, unusual thought content and/or suspicion. Recent reports from the prodromal clinic in Melbourne document a falling trend for conversion rates to psychosis among consecutive annual samples between1995-2005 [20]. This declining rate was correlated with a decrease in the duration of prodromal symptoms prior to clinic intake evaluation. Changes in referral sources may also have increased the rate of “false positive” prodromal subjects, i.e. persons who would not subsequently convert to psychosis anyhow. In a recent study by Yung et al., [21] the psychosis conversion rate at 6 month follow-up is 7%. In a recent review article by Simon et al. [22] of 31 studies the reported transition rates varied from 7 to 54% (mean rate = 24%). Follow-up periods ranged from 6 to approximately 40 months. In a meta-study by Fusar-Poli et al. [9] of 2502 patients there was a consistent transition risk, of 18% after 6 months of follow-up, 22% after 1 year, 29% after 2 years, and 36% after 3 years.

### Controlled clinical trials of treatment in the prodrome

In a recent meta-analysis by Stafford et al. [23] 11 included RCT studies made eight comparisons. The authors identified four trials comparing CBT with supportive counseling and monitoring [24-27]. Two trials compared risperidone and CBT with supportive counseling [28,29]. Further on; one study each compared risperidone and CBT with placebo and CBT [28], olanzapine with placebo [30], integrated therapies with supportive counselling [31] integrated therapies with standard treatment [32] Omega-3 fatty acids with placebo [33] and amisulpride plus a needs based intervention with the needs based intervention alone [34]. All these studies measured conversion to psychosis as the primary dependent variable. The studies demonstrate that treatment (psychosocial, pharmacotherapeutic or both) reduce the conversion rate to psychosis. The McGlashan study demonstrated a significant effect in reducing the severity of positive prodromal symptoms, but also major negative side effects in the form of weight gain. Further, there are preliminary reports indicating a possible effect on conversion rates from psychoeducational family work [35]. These recent studies have contributed to the availability for larger datasets, which suggests that transition to psychosis from a high risk mental state could be preventable. Overall, in the meta-analysis by Stafford et al. [23] the five trials of CBT had a moderate effect on transition to psychosis at both 12 and 18 months. In the sensitivity analyses the effect of CBT on transition remained significant at 12 months. There has also been evidence that complex psychosocial interventions could reduce transition or delay onset of psychosis, relative to supportive counseling or treatment as usual. For the study of Omega-3 fatty acids [33] low quality evidence suggests a beneficial effect for a 12 week course of nutritional supplementation compared with placebo.

### Neurocognitive and structural brain imaging studies in prodromal samples

Neuroimaging and neurocognitive studies have attempted to find reliable psychosis high-risk biomarkers. Two recent meta-analyses at high risk for psychosis showed that high-risk subjects are already neurocognitively impaired relative to matched controls [36,37]. There was a high variability across individual studies, and no reliable neurocognitive biomarkers have adequate sensitivity and specificity for clinical application in individual cases. Across neurocognitive domains, such as executive function, verbal fluency, attention, visual and verbal memory, and working memory, effect sizes were small to medium. Available research confirms that psychosis high risk is characterized by widespread mild cognitive deficits at a level that is intermediate between healthy individuals and those diagnosed with schizophrenia [38] and comparable to those at familial risk [39]. Neurocognitive impairments in psychosis high risk predict longitudinal functioning [40]. The magnitude of neurocognitive deficits is greater in subjects with a psychosis risk state who will later transit to psychosis as compared to those who will not [36], supporting the existence of different psychosis high-risk endophenotypes [36]. Neuroimaging techniques have the potential to find true indicators of psychosis vulnerability [41]. Early studies employed structural imaging methods and focused on regions known to be affected in schizophrenic psychoses such as hippocampus [42,43] or anterior cingulate cortex [44]. Since then, a number of structural studies employing whole-brain methods such as voxel-based morphometry or region-of-interests methods have been published. As compared to matched controls, high risk individuals show significant alterations in regional gray matter volume regardless of whether they subsequently develop the disorder [45]. These alterations are particularly evident in prefrontal and temporolimbic areas [46]. The largest multicenter structural study conducted in UHR showed parahippocampal alterations predicting psychosis onset [45].

### Aims

The primary aim of the current study is to test the effect of a Prodromal Detection and Treatment program at the health care systems level. The study will investigate; 1) If the combination of information campaigns and detection teams modelled will help in identifying individuals at high risk of developing psychosis early, and 2) If a graded, multi-modal treatment program will reduce rates of conversion compared to the rates seen in follow-along assessments. The study will use the knowledge obtained and the treatment strategies developed from recent experimental studies, and test them out on the health care system level as a proof-of-concept study that intervention in the prodromal phase may lead to a reduction in the development of new cases. The endpoint or main outcome will be the rate of conversion to psychosis. The study will be conducted within a translational approach, comprising clinical and genetic sub-projects as well as an FMRI sub study at Stavanger University hospital, Norway done in close collaboration with health regions in Southern Norway. The aim of these sub-projects is to identify neurobiological indicators of psychosis vulnerability.

## Methods/Design

### Overall design

The current study will use a health service design modeled after the TIPS study, using the unique possibilities of the Norwegian catchment area based treatment systems. UHR patients from two areas (the catchment areas of Stavanger and Fonna) will be recruited through information campaigns and assessed by low-threshold detection teams. Recruited individuals will, after giving informed consent, receive structured follow-along by a designated case manager, and receive a multi-modal treatment package containing CBT, family work and Omega-III fatty acids and with the possibility for provision of antipsychotic medication only at imminent risk of conversion. Study personnel blind to treatment allocation will confirm this through a separate symptomatic evaluation.

### Information campaigns and detection teams

Each Prodromal Detection and Treatment site will implement a program building upon the combined experiences from the TIPS study and previous prodromal studies, consisting of Information Campaigns (IC) and Low Threshold Detection Teams (DT) [2]. Prodromal detection, like early psychosis detection, requires active outreach and recruitment using information campaigns and specialized detection teams. As demonstrated by the TIPS project, both elements are likely to be critical [47]. Given that the threshold pathology in the prodrome is likely to be more privately experienced and less publicly apparent, the IC must be intense, persistent, pervasive, and personal. The IC will use three concrete strategies:

A) Teaching the public about early signs of severe mental illness, the importance of getting help early and the existence of the low threshold DTs;
B) Provide targeted education programs for teachers and General Practitioner’s (GPs) about prodromal symptoms and the content and availability of the PDT program;
C) Provide targeted education for clinicians in the specialized mental health services to learn them to recognize possible prodromal individuals.

Potential prodromal individuals in the PDT areas will be referred to a DT by health care providers, educators, or social service agencies or they can be self-referrals in response to the IC. Potential subjects will undergo a telephone and/or a face-to-face screen (Prodromal Questionnaire, PQ-B) [48]. Those who screen positive will be invited to an in-person eligibility and consent evaluation based on the SIPS interview.

### Prodromal treatment package

This will be modeled after practices tried out in the TIPS study as well as practices shown to influence risk of conversion in experimental studies and common to existing prodromal centers. The patients will participate in an individual 2 year follow-along containing the following elements:

1. One-to-one monitoring of clinical status, symptom levels (prodromal and psychotic), risk profiles (suicidality, dangerousness), instrumental and social functioning;
2. One-to-one case management to help deal with clinical, familial, social and vocational crises, needs and deficits;
3. Omega-III fatty acids, in the form of 2g fish oils containing approx. 1.5 g Etyl-Eicosapentaeonic Acid/DHA with 80 mgs Vitamin E per day for 12 weeks;
4. Individual cognitive behavioral therapy (CBT) to deal with social/cognitive distortions and deficits and to maintain real world investment (based on the EDIE II study) [49]. They will be offered 26 sessions of CBT within a six months period. The CBT sessions will be based on established cognitive models, be collaborative, problem orientated, formulation driven, normalizing, educational and time-limited with Socratic questioning/ guided discovery;
5. Individuals that experience functional loss will in addition receive single-family psycho-education to inform patients and families about current problems, how to understand and cope with them, especially within the family [50,51];
6. Anti-anxiety agents and anti-depressants will be available if the patient is so symptomatic that they otherwise would be prescribed these agents by their GPs;
7. Antipsychotic medication will be available if the patient either enters the study with any SIPS positive symptom score at the level of 5, or if any positive prodromal symptom score(s) moves from a level of 3 or 4 to a 5. Use will be open labeled based on the patients’ current symptom profile. The type and dose of medication will be reviewed by an independent clinical practice safety monitoring board.

### Questionnaires and interviews

Participants will be screened with the Structured Interview for DSM-IV (SCID) [52] /(Kiddie SADS for adolescents who are 13–17 years old) and the SIPS at study entrance. All participants will be followed with a symptomatic assessment (SIPS) done monthly for the first six months and then every three months for the last two years. The participant will be evaluated by a designated nevopsychological protocol at baseline, 12 and 24 month follow-up and offered to participate in a fMRI substudy. Assessors will not be part of the treatment teams.

### Prodromal inclusion and exclusion criteria

Start of study inclusion is from March 1^st^ 2012, ending inclusion December 31^st^ 2016.

1) The patient is listed in the national register and residing in the catchment areas of: Stavanger and Fonna; 2) Between 13 and 65 years; 3) Meet diagnostic criteria for prodromal syndrome SIPS criteria [11]; 4) Does not meet current or life-time criteria for any psychotic disorder; 5) The symptoms are not better accounted for by an axis I, axis II or substance use disorder with the exception of schizotypal personality disorder (the presence of any of these disorders in itself is not an automatic reason for exclusion); 6) Does not use any antipsychotic medication currently and have not used antipsychotic medication (regardless of dosage) for more than four weeks lifetime; 7) No known neurological or endocrine disorders that may have caused the presenting psychotic symptoms; 8) The patient is not mentally retarded with an IQ below 70; 9) The patient must be able to understand and speak Norwegian; 10) The patient must be able to understand and sign an informed consent or assent for minors’ document.

### Power calculation

There are no previous studies giving any clear suggestion of the number of high-risk individuals that can be recruited from a well-defined catchment area sample like the current, but previous studies in Melbourne indicate that around 20% of first episode patients have been in contact with the PACE clinic (McGorry, personal communication). Studies up to now give a conservative estimate of a 20% transition rate over the first two years for a non-treated high-risk sample and subsequently a 10% risk in the active treatment group. A power analysis has shown that in order to give an achieved significant reduction of transition rates in the study population with the proposed sample size for the two groups of a) 750 000 with predicted annually detection of 150 first-episode psychosis (FEP) (Bergen and Østfold) and b) 440 000 with annually predicted reduction to 90 FEP (Stavanger and Fonna) through prodromal detection and intervention, the study will have power of 88.9% to yield a statistically significant result.

### The psychosis incidence study

The main outcome of the intervention will be the incidence of psychotic disorders in the participating areas. This will be assessed using the same research diagnostic criteria; in all participating areas over all study years.

#### Incidence study inclusion criteria

1) The patient is listed in the national register and residing in the catchment areas of Stavanger, Fonna, Bergen, and Østfold; 2) Between 13 and 65 years; 3) Meet diagnostic criteria in DSM-IV for first-episode schizophrenia, schizophreniform disorder, schizoaffective disorder, delusional disorder, brief psychotic disorder, affective psychoses (Bipolar I disorder, Bipolar II disorder with psychotic symptoms, major depressive disorder with psychotic symptoms) or psychotic disorders NOS; 4) The patient is (or has recently been) active psychotic with symptoms of delusions, hallucinations, disturbed thinking, unsuitable/ bizarre behaviour which cannot clearly be explained by organic reasons. The symptoms must have lasted the whole day for several days or several times a week for several weeks, not limited to some brief moments corresponding to a score of at least 4 on one or more of the following positive and negative symptom scale (PANSS) [53] symptoms: P1 (delusions), P3 hallucinations), P5 (grandiose thinking), P6 (suspiciousness) and G9 (unusual thought content); 5) This is the first episode of the condition that is being adequately treated. i.e. the patient has not received antipsychotic treatment corresponding to 75% of a defined daily dosage for more than eight weeks (shorter if the symptoms remit); 6) There are no known neurological or endocrine disorders that may have caused the presenting psychotic symptoms; 7) The patient is not mentally retarded with an IQ below 70; 8) Able to understand and speak Norwegian; 9) Able to understand and sign informed consent/assent for minors’ document.

#### Assessments

The primary measure of the incidence study is psychotic caseness as ascertained by SCID (Kiddie SADS for adolescents who are 13–17 years old) assisted by the PANSS interview.

### Prodromal patient neuroimaging protocol

A fMRI protocol is set up for the prodromal study providing the possibility to study prodromal patients, and normal controls. The MR imaging is performed with a 1.5 tesla scanner (450 Discovery; GE Medical Systems) equipped with a standard head-and-spine (HNS) coil.

#### Structural images

A structural image is acquired after the functional imaging using a BRAVO sequence (GE Healthcare); a T1-weight 3D IR-prepared spoiled gradient echo-sequence with the following parameters: TR/TE/TI = 7.9 ms/3.1 ms/450 ms, FOV = 24 × 19.2 cm. Number of slices is 180 with a thickness of 1 mm. The in-plane sampling matrix is set to 240 × 192 resulting in an iso-tropic resolution of 1 mm. The total scan time is 6 min. 3 s.

#### Functional magnetic resonance imaging

##### Working memory task

Participants undergoes fMRI scanning while performing a numeric n-back WM task as used in previous studies [54-56]. The task contains two conditions: (1) in the “2-back” condition, participants are required to press a button when the number they see equales the number seen two numbers before; and (2) in the “0-back” condition, participants have to respond with a button press each time they see the number zero. Numbers between 0 and 9 is displayed for 500 ms with an intertrial interval of 900 ms. Each block consist of 22 stimuli containing three targets and is indicated by an instruction cue displayed for 2 seconds before each block. Stimulation blocks and resting periods alternates within the experiment with a total of six 2-back and six 0-back blocks. During resting periods, volunteers are instructed to fixate on a cross in the center of the screen.

MRI is performed using gradient-echo echo-planar imaging (TR, 2600 ms; TE, 35 ms; flip angle, 90°; matrix, 64 × 64; voxel size, 2 × 4 × 5 mm). The combination of a stimulus onset asynchrony of 1400 ms (500 ms stimulus duration; 900 ms intertrial interval) and a TR of 2600 ms resultes in 13 possible time points of stimulus presentation per TR. Across multiple stimulus presentations, this yields an effective sampling rate of 5 Hz. Twenty-four slices approximately parallel to the bicommissural plane (anterior commissure–posterior commissure plane) are collected, covering the whole brain. Twenty fMRI volumes are acquired per block: 12 during stimulation (2-back or 0-back) and eight during the resting period. Blocks are presented alternately three times in each of the two runs (A B A B A B). A total of 252 volumes are collected.

##### Resting state task

Subjects undergoes one 8 min scan during which they performed a resting-state, low-level baseline task (eyes open, visual fixation on a hair-cross-centered in the screen). MRI is performed using gradient-echo echo-planar imaging. The experiment consisted of a single session during which 180 BOLD sensitive EPI volumes are acquired with 25 axial slices (TR, 2500 ms; TE, 40 ms; flip angle, 90°, 5 mm slice thickness, no interslice gap, matrix, 64 × 64; field of view: 240 × 200 mm^2^; voxel size, 2 × 4 × 5 mm).

##### Dichotic listening task

The paradigm consist of dichotic presentations of pairs of consonant–vowel (CV-) syllables, i.e. two different syllables were simultaneously presented, one to the left and one to the right ear [57-59]. The syllable pairs is formed combining the six syllables /ba/, /da/, /ga/, /pa/, /ta/, and /ka/ to all possible 30 pairs of unidentical syllables (e.g., /ba/ presented to the left and /da/ presented to the right ear), i.e. also including the reversed pairing (e.g., /da/ presented to the left and /ba/ presented to the right ear). The syllables is spoken by an adult Norwegian male voice with constant intensity and intonation. The syllables in each pair are temporally aligned to achieve simultaneous onset of the initial consonants. The stimulus duration varies between 400 and 450 ms. The fMRI paradigm is presented as block-design, including in total nine task blocks (interleaved with a rest block) each containing ten syllable pair presentations, amounting to in total 90 stimulus presentations (equivalent to three times the total set of syllable pairs). The first three blocks are presented with no specific attention instruction (NF), i.e. the subjects are instructed to report the syllable which they heard best in each trial. For the remaining six blocks the subjects are asked to focus their attention to and report the left- (FL) or the right-ear stimulus (FR), respectively. In all three conditions, the instruction is to accurately report the syllable (with no emphasis on response speed). The order of the three FL and the three FR blocks are pseudo-randomised (order: ABBABA). This approach of starting with the NF blocks, followed by the forced attention blocks, is chosen to avoid “carryover” effects that might result from presenting the forced attention conditions first, since individuals might not be able to “not attend” once instructed to attend to a particular side in auditory space [60].

Before entering the MR scanner all subjects conducts five practice trials (with the NF instruction) in order to familiarize them with stimulus material and procedure. Here, the subjects are also informed that in addition to the just-practiced NF condition, two other conditions will be presented, during which they will be asked to selectively attend to one ear and only report the syllable presented to this ear. Inside the scanner, instructions are given via head-coil mounted goggles (Nordic Neuro Lab, Bergen, Norway). Each instruction consists of a brief sentence asking the subject to report the syllable which is heard the best (NF), in the right ear (FR), in the left ear (FL), or to relax (rest block). The instruction screen is after 2500 ms replaced by a fixation cross on which the subjects are instructed to focus their eyes. Throughout all blocks, the inter-stimulus interval is 5500 ms. Stimulus administration is controlled by E-Prime software (Psychology Software Tools Inc., Pittsburgh, PA) and the dichotic stimuli is presented using MR-compatible headphones (Nordic Neuro Lab, Bergen, Norway). The subjects’ response were given orally and is recorded with an mp3-recorder connected to an MR-compatible microphone.

The resulting recordings will be analyzed and coded. The percentage of correctly reported syllables will be determined separately for the left-ear (LE) and the right-ear (RE) stimuli in each of the three conditions. In order to quantify the effect of attention we will calculate two “attentional gain scores” [61]: (1) the increase in the number of correct right-ear report from NF to FR (defined as: REgain = FR_RE − NF_RE); and (2) the increase of correct left-ear report from NF to FL (LEgain = FL_LE − NF_LE).

Functional imaging is performed using a sparse-sampling echo-planar imaging (EPI) sequence, i.e. EPI volumes are acquired with a repetition time of 5.5 s and an acquisition time of 1.5 s, leaving a silent gap of 4 s, during which the dichotic stimuli are presented and the subjects’ verbal response is given [62]. The experiment consists of a single session during which 180 BOLD sensitive EPI volumes were acquired with 25 axial slices (field of view 240 × 75 mm^2^; scan matrix 64 × 64; 3 mm slice thickness, no interslice gap; voxel size 1 × 4 × 3 mm, echo time of 35 ms), covering most of the cerebrum. The experimental stimulation follows a block design with nine blocks, three per condition (see above). Each block contains ten volume acquisitions, with one trial per silent gap and lasting for 55 s. Each block of the nine experimental blocks is followed by a rest-block (10 scans, 55 s).

### Ethical approval

The study is approved by the National Committee for Medical and Health Research Ethics (2009/949). Participation in the prodromal study will be based on written informed consent. All data from the Patient Record System for patients registered in the incidence study will be depersonalized by health professionals who already have access to the information before it is delivered to the researchers.

## Discussion

Evidence has been accumulating that it may be possible to achieve prevention in psychotic disorders. Solid evidence has come forward from the TIPS study for tertiary and secondary prevention, providing proof of concept that prevention in psychosis is possible and might be extended to the disorder itself. Direct but preliminary evidence for primary prevention has been generated by clinical trials in Australia, Europe and the US. If we can identify people about to become psychotic with high accuracy, we can track them to understand more about how psychosis unfolds. Appropriate intervention at this stage could also prevent or delay the onset of psychosis and/or subsequent deterioration, i.e., social and instrumental disability, suicide, aggressive behavior, affective- and cognitive deficits. However, there are still major methodologically and practical challenges that need to be solved. The health services need to be able to identify and recruit persons who meet the current criteria which might be difficult because individuals in the high risk group don’t necessarily seek guidance actively. Other challenges concern the sensitivity and specificity of the current UHR criteria, which in turn reflects the heterogeneity of a psychosis high-risk state as well as the heterogeneity of the traditional mental disorders.

## Abbreviations

UHR: Ultra High Risk
APS: Attenuated Positive Symptom
GRD: Genetic Risk and Deterioration
CBT: Cognitive Behavioral Therapy
RCT: Randomized Controlled Study
SCID-i/P: Structured clinical interview for DSM IV Axis I Disorders
SIPS: Structured Interview for Prodromal Syndromes
PDT: Prodromal Detection and Treatment
TIPS: Treatment and Intervention in Psychosis
CAARMS: Comprehensive Assessment of At-Risk Mental States
NAPLS: North American Prodromal Longitudinal Study
CBT: Cognitive Behavioral Therapy
GP: General Practitioner
FEP: First Episode Psychosis
PANSS: Positive and Negative Symptom Scale
fMRI: Functional Magnetic Resonance Imaging
